# Pronounced inter-gilt variability in the secretory activity of day 11 porcine embryos

**DOI:** 10.3389/fvets.2026.1829423

**Published:** 2026-05-05

**Authors:** Morgan S. Clemens, Claire Stenhouse

**Affiliations:** Department of Animal Science, Pennsylvania State University, University Park, PA, United States

**Keywords:** embryo, maternal recognition of pregnancy, pig, pre-implantation, sex differences

## Abstract

Early porcine embryos undergo rapid growth and extensive physiological remodeling during the peri-implantation period. Despite the importance of this developmental window for establishment of pregnancy, the degree of biological variation among embryos and animals during this stage remains poorly characterized. The objective of this study was to evaluate secretory activity of porcine embryos on gestational day (GD) 11 and to determine the relative contribution of embryonic sex compared with inter-animal variability. Gilts (*n* = 3) were euthanized and hysterectomized on GD 11, embryos were recovered, individually cultured for 24 h, and subsequently sexed by polymerase chain reaction. Concentrations of estradiol and interleukin-1β (IL1B) in spent culture media were quantified by ELISA, and the expression of interleukin-1β2 (*IL1B2*) mRNA was assessed by quantitative PCR. Differences in estradiol and IL1B concentrations in media (*p* ≤ 0.0001), as well as in the embryonic expression of *IL1B2* mRNA (*p* ≤ 0.0001) were observed among gilts. In contrast, there was no effect of embryonic sex on estradiol or IL1B concentrations in media, or embryonic *IL1B2* mRNA expression (*p* > 0.10) in the current dataset. These results demonstrate substantial inter-animal variation in secretory activity of GD11 porcine embryos. The magnitude of this variability exceeded any detectable effect of embryonic sex and highlights the dynamic and heterogeneous nature of early embryo development in pigs. Consideration of this biological variation should be of high importance when designing and interpreting studies investigating early conceptus physiology.

## Introduction

Early pregnancy in the pig is characterized by rapid conceptus (embryo and associated extraembryonic membranes) growth and extensive physiological remodeling as the conceptus increases surface area to prepare for implantation (1–6). Between approximately gestational day (GD) 10 and GD 12, porcine conceptuses undergo dramatic morphological and functional changes that are essential for the establishment and maintenance of pregnancy (1–3, 7, 8). During this period, embryos secrete signaling molecules that interact with the maternal endometrium to regulate maintenance of the corpus luteum and continuation of pregnancy (8–12).

The peri-implantation period in pigs is characterized by some of the most rapid morphological changes observed during mammalian embryonic development. Conceptuses transition from spherical blastocysts to elongated filamentous structures within a short period of time (1, 2, 5, 6). During this transition, conceptuses display distinct morphological stages including spherical, ovoid, tubular, and filamentous forms as elongation proceeds rapidly between GD10 and GD12. Conceptus elongation involves dramatic increases in surface area primarily driven by coordinated remodeling and migration of trophectoderm and extraembryonic endoderm cells, rather than by increases in cell number (1, 5). This rapid expansion of conceptus surface area allows extensive contact with the uterine luminal epithelium and facilitates the exchange of signals and nutrients between the conceptus and the endometrium during the establishment of pregnancy.

Porcine embryos produce signaling molecules that are central to embryo–maternal communication during early pregnancy. Estradiol has long been described as the primary signal involved in maternal recognition of pregnancy in pigs (9, 11, 13–15), although more recent work indicates that this process involves the interactions of multiple conceptus-derived factors (3, 8, 16). Among these, interleukin-1β (IL1B) is an important cytokine that contributes to conceptus elongation and alterations in of the uterine environment during early pregnancy (3, 10, 11, 17, 18). As secretion of these signaling molecules is closely associated with conceptus growth and development, differences in embryo developmental stage may contribute to variation in conceptus secretory activity during the peri-implantation period.

In addition to developmental variation, differences between male and female embryos have been reported in several species. Across mammals, fetal sex influences growth, with male offspring typically exhibiting greater birth weight than females (19–24). Evidence suggests that these differences can arise early in development, with sex-related variation in growth detectable early during gestation. In pigs, male embryos have been reported to be larger than female embryos as early as GD10 (25), and sex-specific differences in placental development and uterine responses have been described later in gestation (26–32). Evidence from other livestock species further suggests that embryonic sex can influence signaling processes during the peri-implantation period. In cattle, female embryos have been shown to produce greater amounts of interferon tau, the primary maternal recognition of pregnancy signal in ruminants, compared to male embryos when cultured *in vitro* (33, 34). These findings suggest that embryonic sex may influence conceptus signaling during the maternal recognition of pregnancy period. Despite increasing evidence for sex-specific differences in placental function later in gestation, comparatively little is known about whether such differences are established during the pre- and peri-implantation stages of pregnancy in the pig.

Despite the appreciation of substantial changes in morphology and function during this period of development, there remains a limited understanding of the extent to which biological variability among embryos and litters contributes to differences in conceptus physiology during the pre-implantation period or how this variability compares with potential effects of embryonic sex. Understanding the magnitude of this variation is important for interpreting studies of early embryo development and embryo–maternal signaling.

Therefore, the objective of this study was to characterize the secretion of estradiol and IL1B, two molecules essential for the establishment of pregnancy, by GD11 porcine embryos and to evaluate the relative contribution of embryonic sex compared with inter-litter variability in these measures.

## Materials and methods

All experimental procedures followed the Guide for the Care and Use of Agriculture Animals in Research and Teaching and were approved by the Institutional Animal Care and Use Committee of Pennsylvania State University (PROTO202402691).

### Experimental animals and sample collection

On their third estrous cycle, gilts (*n* = 3) from the same herd were synchronized by oral supplementation with Matrix (Altrenogest. 6.8 mL daily; Merck) for 14 days. Following withdrawal of Matrix, gilts were observed for signs of estrus. Artifical insemination was performed with semen from the same boar upon detection of estrus behaviors (designated Day 0) and 12 h later. Gilts were euthanized and hysterectomized on day 11 of gestation. The uterine horns were flushed with 15 mL of phosphate buffered saline (PBS, pH 7.5.) and the uterine flushings were collected in a collection dish. Pregnancy status was confirmed by the presence of normally developed embryos. The embryos were transported to the lab and embryos were individually cultured for 24 h in 24-well plates containing 750 μL of RPMI-1640 medium (Sigma Aldrich, R8755) with 5% charcoal-stripped fetal bovine serum (Gibco, 12,676–029) and 1% antibiotic antimycotic solution (Sigma Aldrich, A5955) at 38.5 °C. Following the culture period, each embryo was snap-frozen in liquid nitrogen, and the culture media was stored at −80 °C for analysis.

### Extraction of DNA and RNA from embryos

DNA and RNA were extracted from the embryos using an AllPrep DNA/RNA Micro Kit (Qiagen, 80,284) according to the manufacturer’s protocols. The RNA and DNA were quantified spectrophotometrically (NanoDrop ND-1000 Spectrophotometer) and stored at -80 °C until required.

### Sexing of embryos by polymerase chain reaction

Polymerase chain reaction (PCR) was performed using extracted DNA to assess the presence of the sex-determining region Y (SRY) gene to determine embryonic sex. PCR was also performed on each embryo for Zinc Finger Protein X-Linked (ZFX) which is expressed by both male and female embryos as a positive control. Primer sequences were previously validated for sexing porcine embryos (35) and are described in Supplementary Table S1. No template controls containing no DNA were included as negative controls. The PCR reaction was performed using ExtremeTaq HiFi Red Mix (Azura Genomics, AZ-1911) in 200 μL PCR tubes. Each reaction consisted of 12.5 μL of ExtremeTaq HiFi Rex Mix, 1 μL of forward primer (10 μM), 1 μL of reverse primer (10 μM), and 25 ng of DNA. Nuclease free water was added to the reaction for a total reaction volume of 25 μL. The reaction was incubated using a Thermal Cycler (SimpliAmp, Applied Biosystems). The initial denaturation and enzyme activation stage were performed at 95 °C for 2 min. Following this, 35 cycles of incubation were performed at 95 °C for 15 s (denature), 60 °C for 15 s (annealing), and 72 °C for 30 s (extension). The resulting PCR product was analyzed using agarose gel electrophoresis. The gel was prepared to contain 1.2% agarose (Invitrogen, 16,500–100) in 1X tris-acetate EDTA (TAE) buffer. A 50 bp DNA ladder was used (Azura Genomics, AZ-1151) and the gel was imaged using a ChemiDoc XRS + system (BioRad).

### Quantification of the expression of *IL1B2* mRNA

RNA was isolated using an AllPrep DNA/RNA Micro Kit as described above. The SuperScript III First Strand cDNA Synthesis System (Invitrogen, 18,080,051) was used to synthesize complementary DNA (cDNA). Each reaction consisted of 50 ng of RNA, 1 μL of Oligo DT, 1 μL of dNTP, and nuclease free water for a total reaction volume of 10 μL in a 200 μL tube. Representative samples from each gilt were used as no reverse transcriptase (NRT) negative controls to check for genomic DNA contamination. The samples were incubated at 65 °C for 5 min and then cooled to 4 °C using a Thermal Cycler (SimpliAmp, Applied Biosystems). Then, 2 μL of 10X RT buffer, 4 μL of 25 mM MgCl_2_, 2 μL of 0.1 M of DTT, and 1 μL of RNaseOUT (40 U/μL) were added to each reaction. Superscript III enzyme (1 μL) was added to each of the samples, while 1 μL of RNase free water was added to each NRT reaction. The reactions were incubated at 50 °C for 50 min and then 85 °C for 5 min. RNase H (1 μL) was added to each tube and incubated at 37 °C for 20 min. The resulting cDNA was stored at −20 °C until required.

The expression of interleukin 1 beta 2 (*IL1B2*) mRNA was quantified by quantitative polymerase chain reaction (qPCR). Primer information can be found in Supplementary Table S2. Primers were validated by performing an 8-point 1:2 serially diluted standard curve (starting at a 1:5 dilution) on a representative pool of cDNA from all samples. All primers had an efficiency between 90 and 110% and an R squared ≥ 0.990. Each reaction consisted of 5 μL PowerUp SYBR Green Master Mix (Applied Biosystems, A25742), 1 μL forward primer (10 μM), 1 μL reverse primer (10 μM), and 1 μL ultrapure water. Each reaction contained 2 μL of cDNA, except for the no template controls (NTCs), in which 2 μL of nuclease free water was added. Each reaction was run in triplicate using a QuantStudio 3 qPCR machine (Applied Biosystems). Thermal cycling conditions consisted of 40 cycles beginning with 50 °C for 2 min, 95 °C for 2 min, 95 °C for 15 s, and 60 °C for 1 min. Beta-actin (*ACTB*) was quantified, and the expression was unaffected by gilt or embryonic sex (*p* > 0.05) and therefore was utilized as a reference gene. The relative expression of *IL1B2* mRNAs was quantified using the ΔΔCt method normalizing to *ACTB* expression.

### Quantification of estradiol and interleukin 1β in culture media

Estradiol (Eagle Biosciences, ESD31-K01) and interleukin-1β (IL1B; Abcam, AB100754) concentrations in spent culture media were quantified using enzyme-linked immunosorbent assays (ELISAs) according to the manufacturers’ instructions. All samples, standards, and controls were assayed in duplicate. Plate absorbance was measured at 450 nm using a spectrophotometer (Cytation 5, BioTek).

For the estradiol ELISA, the lower detection limit was 10 pg./mL, with an intra-assay coefficient of variation of 1.54% and an inter-assay coefficient of variation of 4.95%. For the IL1B ELISA, the assay sensitivity was 6 pg./mL, with an intra-assay coefficient of variation of 2.18% and an inter-assay coefficient of variation of 13.54%.

### Statistical analysis

ELISA and qPCR data were analyzed and visualized using GraphPad Prism (version 10.6.1). For the estradiol ELISA, the mean of each duplicate was calculated, and concentrations were determined from a four-parameter logistic standard curve. For the IL1B ELISA, the mean of each duplicate was calculated, and concentrations were determined using logarithmic linear regression. A ROUT outlier test was applied and showed no outliers. Data were assessed for normality using the Shapiro–Wilk test and were considered normally distributed when *p* > 0.05.

For the expression of *IL1B2* mRNA, the mean of each technical triplicate was calculated and normalized to the expression of *ACTB* mRNA using the ΔΔCt method. Outliers were identified and removed using the ROUT outlier test. Specifically, in gilt 1, 2 males and 1 female were removed. In gilt 2, 3 males and 0 females were removed. In gilt 3, 1 male and 1 female were removed. Data were assessed for normality using the Kolmogorov–Smirnov test and were considered normally distributed when *p* > 0.05.

**Figure 1:**
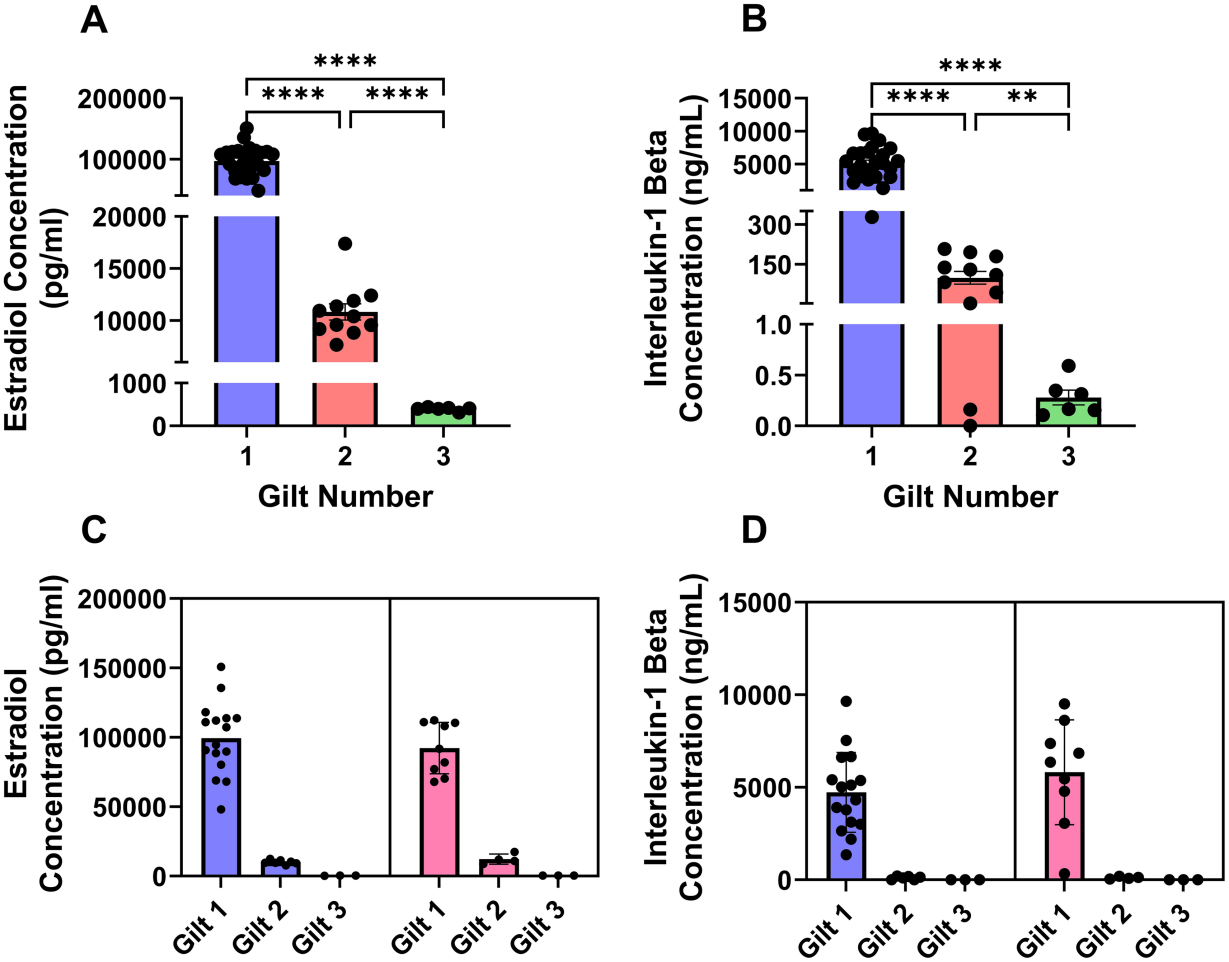
Estradiol **(A, C)** and IL1B **(B, D)** concentrations in spent culture media. Panels A and B show concentrations by gilt. Panels C and D show the same data stratified by embryonic sex, with individual gilts displayed within each sex. Points represent individual samples; bars represent mean ± SEM.

**Figure 2:**
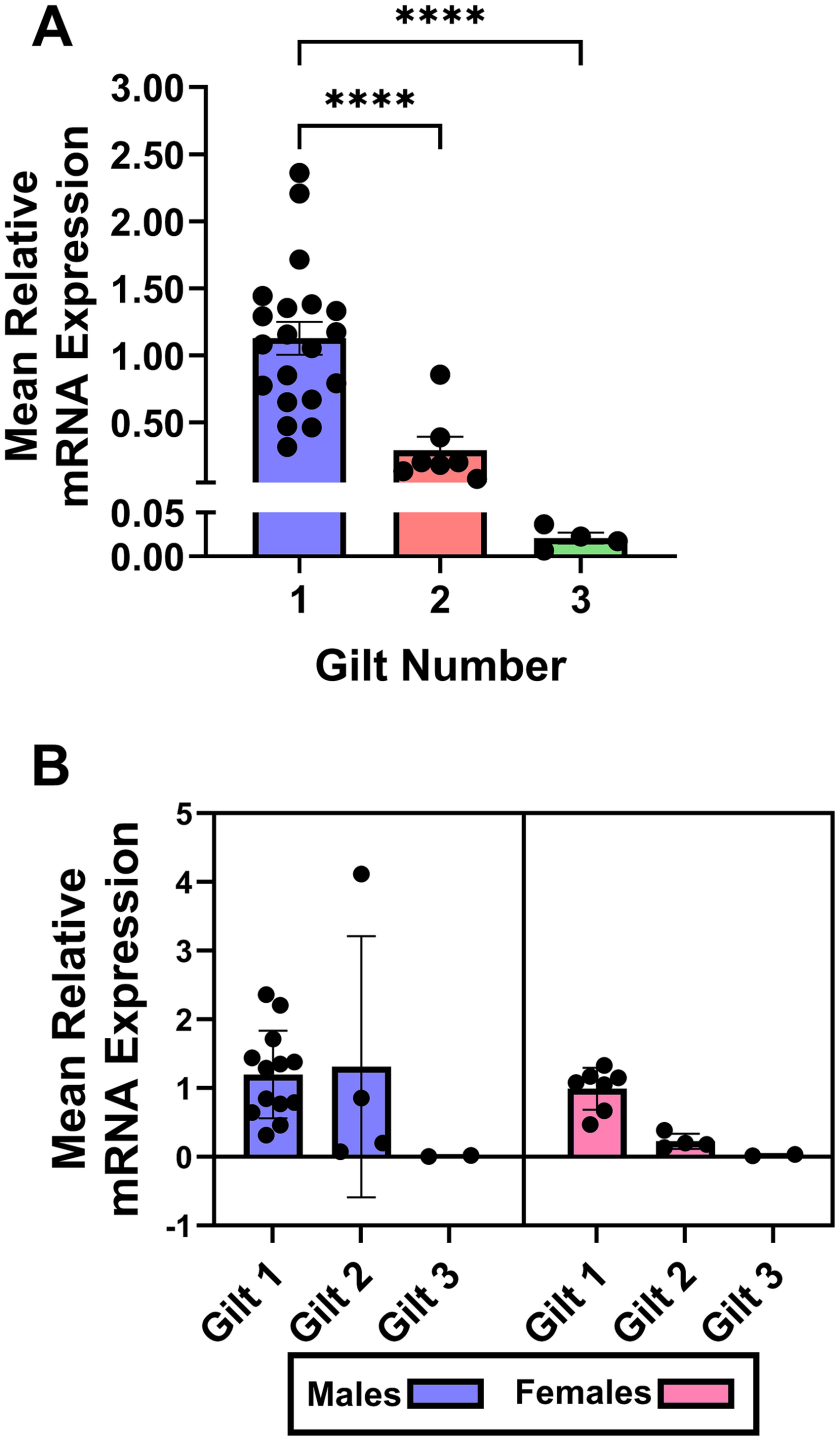
Expression of *IL1B2* mRNA in cultured embryos. **(A)** Relative mRNA expression across gilts. **(B)** Relative mRNA expression separated by embryonic sex (males and females), with individual gilts shown within each sex. Points represent individual samples; bars represent mean ± SEM.

For comparisons among gilts, a Welch’s ANOVA test was applied (Figures 1A,B, 2A). For comparisons within gilts, a Welch’s *t*-test was applied (Supplementary Figures S1, S2). A nested t-test was applied with gilt as a random effect to account for biological variation between gilts (Figures 1C,D, 2B).

Statistical significance was defined as *p* ≤ 0.05. Values between 0.05 and 0.10 were considered as tendencies towards significance, and values > 0.10 were considered not significant.

## Results

### Variation in embryo yield and sex ratios among gilts

**Figure 3:**
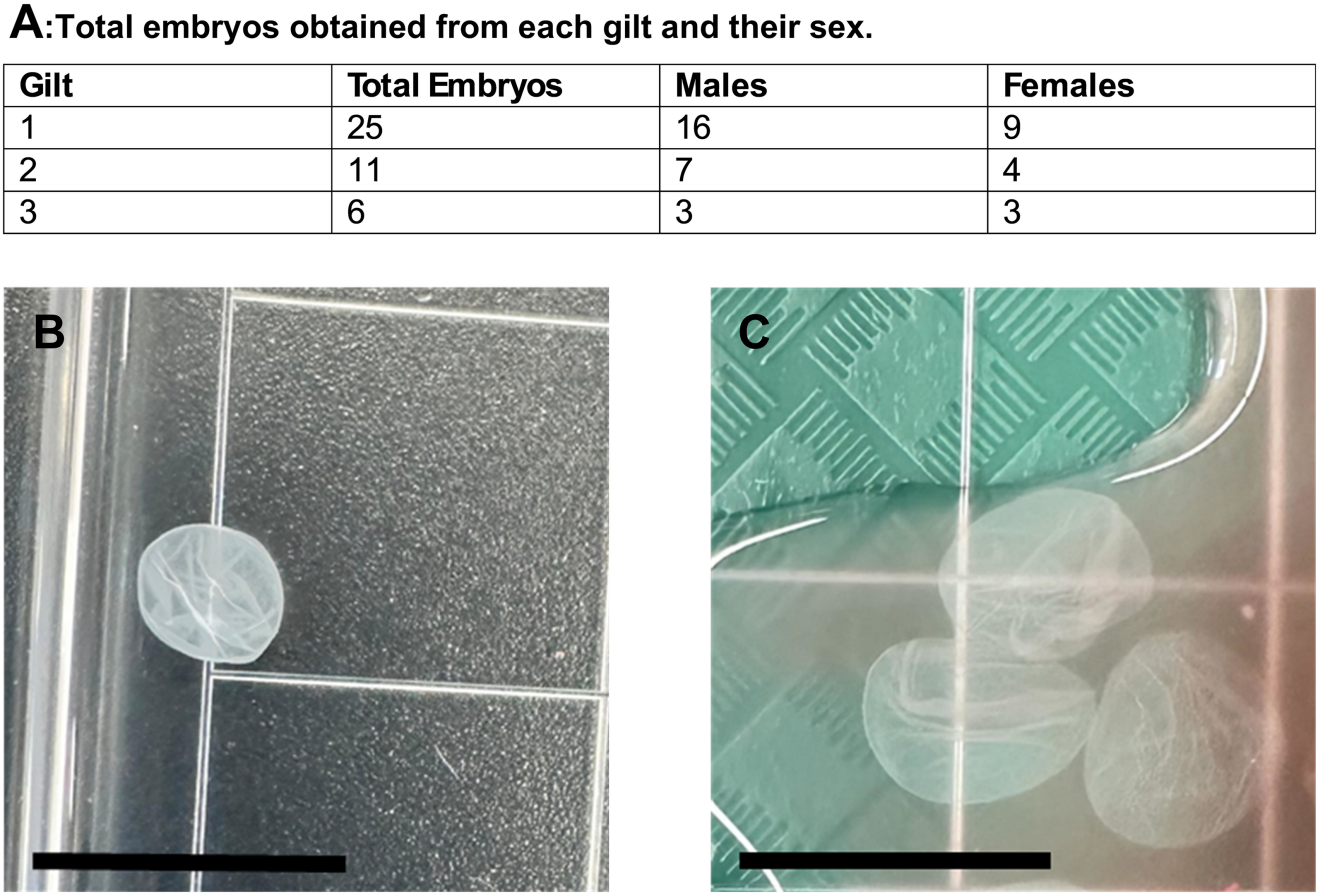
**(A)** Total number of embryos recovered from each gilt, with sex determined by PCR. **(B–C)** Representative images of embryos from Gilt 1 **(B)** and Gilt 3 **(C)**. Scale bar = 15 mm.

The number of embryos recovered varied among gilts (Figure 3). Gilt 1 yielded the greatest number of embryos, with 16 males and 9 females. From gilt 2, 7 male and 4 female embryos were recovered, whereas the fewest embryos were recovered from gilt 3, with 3 males and 3 females. In total, 42 embryos were recovered from the three gilts, comprising 26 males and 16 females.

All embryos exhibited spherical morphology. Although individual embryo dimensions were not recorded, embryos recovered from gilt 1 appeared larger than those from the other gilts (Figure 3). Embryos from gilt 2 appeared intermediate in size, whereas embryos from gilt 3 appeared to be the smallest.

### Quantification of estradiol and IL1B in conditioned media

Following the 24 h culture period, concentrations of estradiol and IL1B in spent culture media were quantified by ELISA. Concentrations of both estradiol and IL1B differed in spent culture media among embryos recovered from the three gilts (Figure 1A). Estradiol concentrations in media from embryos recovered from gilt 1 were greater than those from embryos recovered from gilt 2 (*p* ≤ 0.0001) and gilt 3 (*p* ≤ 0.0001). Estradiol concentrations were also greater in media from embryos recovered from gilt 2 compared with gilt 3 (*p* ≤ 0.0001).

Similar differences were observed for the concentration of IL1B in the media (Figure 1B). Media from embryos recovered from gilt 1 contained greater concentrations of IL1B than media from embryos recovered from gilt 2 (*p* ≤ 0.0001) and gilt 3 (*p* ≤ 0.0001). Media from embryos recovered from gilt 2 also contained more IL1B than media from embryos recovered from gilt 3 (*p* ≤ 0.01).

There were no significant sex-related differences detected within the present dataset (*p* > 0.10) in the concentration of estradiol or IL1B in the media, both when the comparison was made within gilt (Supplementary Figure S1) or utilizing a nested model to account for having multiple embryos from each gilt (Figures 1C,D).

### Quantification of the embryonic expression of *IL1B2* mRNA

The expression of *IL1B2* mRNA differed among embryos recovered from the different gilts (*p* ≤ 0.0001; Figure 2A). Multiple comparison analysis indicated that embryos from gilt 1 had greater expression of *IL1B2* mRNA than embryos from gilts 2 and 3 (p ≤ 0.0001). Similarly, there was a tendency towards decreased expression of *IL1B2* mRNA in embryos from gilt 3 compared to gilt 2 (*p* = 0.09). There was no effect (*p* > 0.10) of embryonic sex on the expression of *IL1B2* mRNA when compared within gilt (Supplementary Figure S2) or when a nested *t*-test model was utilized (Figure 2B).

## Discussion

Early pregnancy in the pig is characterized by rapid conceptus growth and extensive physiological remodeling between GD10 and GD12, a developmental window during which embryos produce signaling molecules essential for maternal recognition of pregnancy (8). The present study evaluated secretion of estradiol and IL1B by GD11 porcine embryos and examined the relative contributions of embryonic sex and inter-gilt variability to these measures. The principal finding was that secretory activity differed markedly among embryos recovered from different gilts, whereas no significant sex-related differences were detected within the present dataset in estradiol secretion, IL1B secretion, or expression of *IL1B2* mRNA. These observations indicate that biological variability among litters represents a major source of variation in conceptus signaling during this stage of development.

Substantial variability among gilts was evident in both embryo number and secretory activity. Embryos recovered from gilt 1 secreted greater concentrations of estradiol and IL1B and exhibited greater expression of *IL1B2* mRNA than embryos from the other two gilts. Although individual embryo dimensions were not recorded, embryos from gilt 1 appeared larger than those from gilts 2 and 3. Additionally, the number of embryos recovered from each gilt varied greatly, which could indicate that there may have been differences in the uterine environment that may have contributed to differences observed in embryos across gilts. Differences in developmental stage among litters may contribute to these observations. During the peri-implantation period, porcine conceptuses undergo rapid transitions from spherical to elongated morphologies accompanied by extensive cellular remodeling and changes in gene expression (1, 2, 5, 6). As this developmental progression occurs during a short period of time, even small differences in the timing of ovulation or fertilization among gilts could result in embryos at slightly different stages of development when recovered on the same gestational day. Conceptus size and cell number increase rapidly during the pre-implantation period, and secretion of signaling molecules such as estradiol and IL1B increases as embryos develop and prepare for elongation (6, 10). Consequently, relatively small differences in developmental stage both within and among litters may lead to substantial differences in measured secretory activity.

As embryos transition from spherical to elongating stages, conceptus size, cellular activity, and steroidogenic capacity increase, leading to greater secretion of estradiol (1, 4, 6). Therefore, relatively small differences in developmental progression within and among litters recovered on the same gestational day could contribute to the differences in estradiol concentrations observed in the present study. Previous work has demonstrated that estradiol production increases as conceptuses grow from spherical blastocysts toward elongating stages, reflecting increases in trophoblast activity and developmental progression (6). Similarly, IL1B has emerged as an important mediator of conceptus–endometrial communication during early pregnancy, contributing to conceptus elongation and remodeling of the uterine environment (3, 10, 11, 36). It is possible that greater concentrations of both estradiol and IL1B detected in embryos from one gilt therefore may reflect differences in conceptus developmental progression rather than differences attributable to embryonic sex.

Despite reports of sex-associated differences in growth and utero-placental physiology later in gestation, no significant sex-related differences in estradiol or IL1B secretion, or the expression of *IL1B2* mRNA were observed in the present study. Male embryos have been reported to exhibit larger diameters than females as early as GD10 in pigs (25), suggesting that sex-specific differences in developmental rate may emerge early in gestation. However, the current findings indicate that any differences attributable to embryonic sex were smaller than the variability observed among litters in this dataset. A *post hoc* power analysis based on the variability observed in this study indicated that approximately 176 embryos per sex would be required to achieve 80% statistical power to detect differences in estradiol or IL1B secretion between male and female embryos. It is important to note that the small sample size in this study may increase they likelihood of Type 1 errors as embryos from the same gilt are not independent. Due to this, a nested model was applied to reduce the effect of gilt. In pigs, transcriptional divergence between male and female embryos increases progressively during early gestation, with relatively modest numbers of differentially expressed genes reported during the pre-implantation period (37). The limited transcriptional divergence reported at this stage of development in the Zeng et al. (37) study may help explain why detectable physiological differences between male and female embryos were not observed in the present study.

The magnitude of inter-animal variability observed here has important implications for the design and interpretation of studies investigating early conceptus biology. Previous studies have also reported substantial variation in conceptus morphology and development among embryos recovered on the same gestational day (1, 2, 25, 38). Such variability reflects the rapid and asynchronous nature of conceptus development during the peri-implantation period. Consequently, experiments examining embryo signaling during this developmental window should account for litter effects and developmental stage when interpreting results. Analytical approaches that account for the nested biological structure of embryos within gilts may therefore improve interpretation of data obtained during this dynamic stage of development.

## Conclusion

This study demonstrates substantial inter-gilt variability in the secretory activity of GD11 porcine embryos. Differences in estradiol and IL1B concentrations in conditioned culture media, as well as the embryonic expression of *IL1B2* mRNA were substantial among gilts, highlighting the dynamic and heterogeneous nature of conceptus development during the peri-implantation period in pigs. Importantly, these findings highlight the need to account for inter-animal variability when investigating early embryo physiology.

## Data Availability

The original contributions presented in the study are included in the article/Supplementary material, further inquiries can be directed to the corresponding author.

## Supplementary material

SUPPLEMENTARY FIGURE S1 Estradiol **(A–C)** and IL1B **(D–F)** concentrations in the culture media secreted by male and female embryos compared within gilt. Mean values are presented ± SEM.

SUPPLEMENTARY FIGURE S2 Expression of *IL1B2* mRNA by male and female embryos compared within gilt. Statistics could not be performed on Gilt 3 **(D)** due to low sample size. Mean values are presented ± SEM.
