# Supplementary material for: Pronounced inter-gilt variability in the secretory activity of day 11 porcine embryos
Source: Front Vet Sci. 2026 May 5;13:1829423. doi: 10.3389/fvets.2026.1829423 (PMC13183555; doi:10.3389/fvets.2026.1829423)
Supplement: Supplementary file 3 [file Table_1.DOCX]

**Supplementary Table 1: Primers Used for PCR.**

| **Gene** | **Gene Name** | **Accession Number** | **Sequence (5′–3′)** | |
| --- | --- | --- | --- | --- |
| *SRY* | Sex-determining region Y | NC_010462.3 | Forward | GAAAGCGGACGATTACAGCC |
|  |  |  | Reverse | TTGCGACGAGGTCGGTATTT |
| *ZFX* | Zinc finger protein X-linked | NC_010461.5 | Forward | ATAATCACATGGAGAGCCACAAGCT |
|  |  |  | Reverse | GCACTTCTTTGGTATCTGAGAAAGT |
