# Supplementary material for: Pronounced inter-gilt variability in the secretory activity of day 11 porcine embryos
Source: Front Vet Sci. 2026 May 5;13:1829423. doi: 10.3389/fvets.2026.1829423 (PMC13183555; doi:10.3389/fvets.2026.1829423)
Supplement: Supplementary file 4 [file Table_2.DOCX]

| **Gene** | **Gene Name** | **Accession Number** | **Sequence (5′–3′)** | | **Amplicon size (bp)** |
| --- | --- | --- | --- | --- | --- |
| *ACTB* | Beta-actin | DQ845171 | Forward | CACGCCATCCTGCGTCTGGA | 100 |
|  |  |  | Reverse | AGCACCGTGTTGGCGTAGAG |  |
| *IL1B2* | Interleukin-1  beta 2 | NM_001302388.1 | Forward | GCCAATGGTTTTCTCTGTGATGCC | 158 |
|  |  |  | Reverse | CTCATGCAGAACACCACTTCTCTC |  |

**Supplementary Table 2: Primers Used for qPCR.**
